# Effect of Clinoptilolite and Sepiolite Nanoclays on Human and Parasitic Highly Phagocytic Cells

**DOI:** 10.1155/2015/164980

**Published:** 2015-05-27

**Authors:** Yanis Toledano-Magaña, Leticia Flores-Santos, Georgina Montes de Oca, Alfonso González-Montiel, Juan-Pedro Laclette, Julio-César Carrero

**Affiliations:** ^1^Departamento de Inmunología, Instituto de Investigaciones Biomédicas, Universidad Nacional Autónoma de México, Ciudad Universitaria, 04510 México, DF, Mexico; ^2^Centro de Investigación y Desarrollo Tecnológico, S.A. de C.V., Avenida de los Sauces No. 87, Mz 6, Parque Industrial Lerma, 52000 Toluca, Mexico

## Abstract

Nanoclays have potential applications in biomedicine raising the need to evaluate their toxicity in *in vitro* models as a first approach to its biocompatibility. In this study, *in vitro* toxicity of clinoptilolite and sepiolite nanoclays (NC) was analyzed in highly phagocytic cultures of amoebas and human and mice macrophages. While amebic viability was significantly affected only by sepiolite NC at concentrations higher than 0.1 mg/mL, the effect on macrophage cultures was dependent on the origin of the cells. Macrophages derived from human peripheral blood monocytes were less affected in viability (25% decrease at 48 h), followed by the RAW 264.7 cell line (40%), and finally, macrophages derived from mice bone marrow monocytes (98%). Moreover, the cell line and mice macrophages die mainly by necrosis, whereas human macrophages exhibit increased apoptosis. Cytokine expression analysis in media of sepiolite NC treated cultures showed a proinflammatory profile (INF*γ*, IL-1*α*, IL-8, and IL-6), in contrast with clinoptilolite NC that induced lees cytokines with concomitant production of IL-10. The results show that sepiolite NC is more toxic to amoebas and macrophages than clinoptilolite NC, mostly in a time and dose-dependent manner. However, the effect of sepiolite NC was comparable with talc powder suggesting that both NC have low cytotoxicity *in vitro*.

## 1. Introduction

Clinoptilolite and sepiolite clay are zeolites that belong to a complex group of aluminosilicates used for nanocomposites applications [1, 2]. They are used as metal oxides supports, antimicrobials [3], enzyme stabilizers [4], for absorption of heavy metals [5–7], and additives for the development of nanocomposites [8]. Because of this, in the last decade they have attracted increasing interest in biomedicine, mainly, nanoclays (NC) dispersed into polymeric matrices which have been proposed as good candidates for drug delivery systems [9–14], dental adhesives [15], bone tissue engineering [16], and immunosensors [17]. However, their use in humans has been hampered by the insufficient information regarding their safety, and toxicological assessment on* in vitro* and* in vivo* models is absolutely necessary [18–20]. These evaluations are needed because the properties of nanomaterials, such as the surface area, zeta potential, and size, can modify their biological interactions compared to microsized materials [21]. Moreover, it has been reported that toxicity of nanomaterials also depends on the model used [22], emphasizing the need for appropriate methodologies and a unified evaluation [23].

According to the International Agency for Research on Cancer, there are few reports regarding* in vitro* and* in vivo* clinoptilolite and sepiolite NC biocompatibility [24]. In this sense, macrophages can be an appropriate model for* in vitro* cytotoxic studies [25, 26] due to the NC applications as nanovehicles which can reach the bloodstream and other tissues. Macrophages are pivotal cells of the innate immune response, specialized in the scavenging of foreign bodies in mammals and widely used in toxicity assays [27–29]; besides, macrophages are considered one of the most phagocytic cells in mammals. On the other hand, the amoeba* Entamoeba histolytica*, the protozoan parasite causing human amoebiasis, is among the most active phagocytic and proteolytic cells in nature, and it has been used as a model to evaluate toxicity of carbon nanotubes [30]. Even though the two systems are quite far apart (mammal and protozoan, for defense and feeding, resp.), they share the characteristic of being the most active highly phagocytic cells in nature, favoring the uptake of the material and the study of toxicity thereof even at low concentrations. In this work, we evaluated the cytotoxicity of clinoptilolite and sepiolite NC* in vitro* by determining their effect on the viability of macrophages from human, mice, and the RAW 264.7 cell line as well as in* E. histolytica *trophozoite cultures, the type of cell death induced (apoptosis or necrosis), and the cytokine profiles released by treated macrophages, all of them as a first approach to determining clinoptilolite and sepiolite NC biocompatibility.

## 2. Materials and Methods

### 2.1. Characterization of Clinoptilolite and Sepiolite NC

Characterization analysis was performed in the USAI, Facultad de Química, UNAM. To determine the chemical structure of NC, X-ray powder diffraction was obtained in a Bruker diffractometer model D8 Advance, with a copper anode as X-ray source (*K*_*α*1_ = 0.154060 nm); chemical composition was determined using a ICP-ms, Bruker Aurora M90 following the percentage of the enlisted elements Si, Al, Fe, Ca, Mg, Ti, P, Mn, Na, K, and S. Finally, samples after the suspension procedure in culture media (below) were observed in Low Vacuum Transmission Electron Microscopy (TEM) and Scanning Electron Microscopy (SEM). Qualitative and semiquantitative microanalysis was performed to determine the size and shape of nanoclays.

### 2.2. Nanoclays and Talc Suspension

Suspensions of clinoptilolite NC (Valfor-100, Silicatos y derivados S.A. de C.V. Mexico), sepiolite NC (sepiolite powder, Sigma-Aldrich, USA) and asbestos-free talc powder (Talc, tested according to Ph. Eur; Sigma-Aldrich, USA) were obtained by sonication of each NC in culture media four times during 15 s each with amplitude of 50% and a frequency of 130 kHz using a sonicator tip Branson Sonifier, USA. TYIS-33 media supplemented with 10% of adult bovine serum and high glucose DMEM media supplemented with 10% of fetal bovine serum were used for amoeba and macrophages, respectively. This treatment was efficient preventing NC aggregates without affecting particle size (data not shown). Stock suspensions of each NC containing 1000 *μ*g/mL in each media were prepared as mentioned, stored at 4°C, and used for the following experiments.

### 2.3. Parasite Culture and Treatment

*E. histolytica* HM1-IMSS trophozoites were axenically grown at 37°C in TYI-S33 medium supplemented with 10% bovine serum, 3% vitamins (Diamond Vitamin Tween 80 Solution 40x, Sigma Aldrich, USA), and 0.1% antibiotic (Penicillin-Streptomycin 10,000 U/mL, GIBCO, USA). Amoebas (1 × 10^5^/mL) were placed in tubes with supplemented TYI-S33 and added with clinoptilolite or sepiolite NC so that the final volume was 3 mL and the concentrations of clinoptilolite and sepiolite NC were as follows: 10, 100, 500, and 1000 *μ*g/mL.

### 2.4. Amoebic Viability

The viability and morphology of amoebic trophozoites were assessed at 24, 48, and 72 h of coincubation with the NC employing two different methods: (1) the vital marker trypan blue to evaluate viability and (2) the carboxyfluorescein diacetate (CFDA Vibrant kit, Invitrogen, USA) plus propidium iodide to evaluate morphology. In brief, amoebic culture tubes were incubated on ice for 5 min in order to detach the parasites, and 10 *μ*L of Trypan blue 0.4% or 1 *μ*L CFDA 5 *μ*M plus 1 *μ*L propidium iodide 1.5 *μ*M was added to aliquots of 100 *μ*L and incubated at room temperature for 15 min. Viable and dead cells were counted in a fluorescence microscope Olympus BX51 using a haemocytometer. Of six independent experiments each one by triplicate was done for each NC assayed.

### 2.5. Macrophage Culture

Macrophages from three different sources were used: macrophages derived from human peripheral blood monocytes (HMDM), macrophages derived from CD1 mice bone marrow monocytes (MMDM), and the RAW 264.7 cell line. HMDM were derived from monocytes isolated from blood samples of 10 healthy individuals at the Hospital Arnau de Vilanova (Lleida, Spain) with written consent. Monocytes were isolated in a Ficoll gradient and then placed in Petri dishes with supplemented RPMI 1640 medium at 37°C under 5% CO_2_ for 5 days, changing the medium every 48 h for the monocytes differentiation into macrophages.

MMDM were obtained from four-week-age CD1 mice. Once euthanized, the femur and tibia were carefully removed and kept in PBS. After treatment with absolute ethanol for 3 min, the femur and tibia were washed with RPMI 1640 medium supplemented with 10% fetal bovine serum, 1% gentamicin, and 1% of 2-mercaptoethanol. Bone marrow cells were removed from the bones using scissors, washed twice with nonsupplemented RPMI 1640 medium, and followed by erythrocytes lysis. Adherent white blood cells were washed with PBS and suspended in supplemented RMPI 1640 medium. Cells were harvested in Petri dishes with 10 mL of medium and incubated at 37°C and 5% CO_2_, changing the medium every 48 h during 5 days for monocytes differentiation into macrophages. The RAW 264.7 cell line was cultured in RPMI 1640 medium supplemented as described above and maintained at 37°C under a 5% CO_2_. Macrophages were sorted by flow cytometry using a specific F4/80 antibody.

### 2.6. Macrophages Treatment with the NC and Viability Assays

Effect of clinoptilolite and sepiolite NC was determined for the three types of macrophages. For each experiment, 1 × 10^5^ macrophages per well were placed in 96-well plate with 100 *μ*L of supplemented RPMI 1640 and enough NC suspension to reach concentrations of 10, 100, 500, and 1000 *μ*g/mL in each well. RAW 264.7 and MMDM culture treated were incubated for 60 h, whereas HMDM were incubated only during 48 h, taking an aliquot every 12 h for determining viability and death as described below. Of three independent experiments each one by triplicate was done for each NC assayed. There are no reports of the concentrations of clinoptilolite and sepiolite NC to which humans could be exposed; however, the reports where NC toxicity has been analyzed reported concentrations between 1 and 1000 *μ*g/mL.

### 2.7. Transmission Electron Microscopy

Amoebic and macrophage culture treated with each NC were centrifuged at 1800 rpm for 5 min and washed 3 times with phosphate buffer solution (PBS, pH 7.4). Cells were then fixed in 4% formaldehyde and 1% glutaraldehyde in PBS by mixing equal volume of fixative and cell suspension. After centrifugation at 1800 rpm for 10 min, the pellet was kept in fresh fixative overnight. Then, the cells were treated 3 times for 15 min with 8% (0.2 M) sucrose in PBS after fixation with 1% OsO4 in PBS for 1 h and rinsed with PBS for 30 min. For dehydration process, ethanol solutions (50, 70, and 95%) were added to the pellets for 15 min each one, absolute ethanol for 15 min twice, and 100% of propylene oxide for 30 min. Infiltration was done with LR white resin (Ted Pella Inc., USA), first adding 1 : 1 LR White: Propylene Oxide for 2 h to the pellets and then stored overnight in 2 : 1 LR White: Propylene Oxide. Samples were embedded in gelatin capsules and baked in 60°C oven for 48 h. Ultrathin sections of 0.5 *μ*m were collected on Formvar/Carbon 200 mesh and Nickel grids and stained with uranyl acetate for 15 min and lead citrate for 3 min.

### 2.8. Viability, Apoptosis and Necrosis

The viability, apoptosis, and necrosis of macrophages treated with NC were determined every 12 h using the Annexin V-FITC Apoptosis detection kit I (BD Pharmingen, USA), according to the protocol of BD Pharmingen. Briefly, for each time point, the culture medium of each well was placed in an Eppendorf Tube and stored at −20°C until use for cytokine analysis, and the wells were refilled with 100 *μ*L of PBS. Annexin V and propidium iodide markers were added and the cells further incubated for 15 min. After the incubation time, the cells were fixed with 50 *μ*L of a stock solution of p-formaldehyde 3.7%, and the samples were read in a FACS Canto flow cytometer (Becton Dickinson, USA).

### 2.9. Th1, Th2, and Treg Cytokines Determination

The cytokine secretion pattern of macrophages incubated with clinoptilolite or sepiolite NC was determined in the supernatant of cultures at 24, 36, and 48 h after coincubation. The expression of GM-CSF, IFN-*γ*, IL-1*α*, IL-2, IL-4, IL-5, IL-6, IL-10, IL-17, and TNF-*α* was measured using the mouse and human Th1/Th2 10plex FlowCytomix Multiplex kit (eBioscience, USA) according to the provider protocol. Briefly, marked beads were added to the supernatant, and after incubation for 15 min the cytokines were measured in a FACS Canto flow cytometer.

### 2.10. Statistical Analysis

Data were analyzed with a Two-way ANOVA (*P* < 0.05) followed by a Tuckey post hoc test (*P* < 0.05) (Microsoft Excel, 2010).

## 3. Results

### 3.1. Characterization of Clinoptilolite and Sepiolite NC

**Figure 1:**
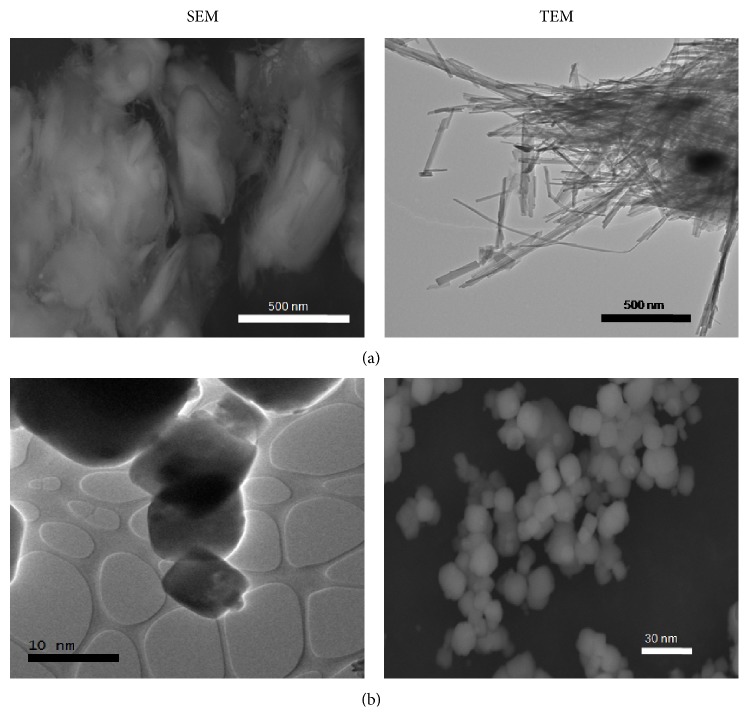
Transmission and scanning electron microscopy of sepiolite and clinoptilolite nanoclays prepared in the DMEM culture media. Sepiolite nanoclays are fibers with a diameter size down to 20 nm (a), whereas clinoptilolite nanoclays are deformed octahedrons with a size down to 30 nm (b).

X-ray powder diffraction pattern of nanoclays showed a typical distribution of diffraction planes associated with the sepiolite and clinoptilolite zeolites. Sepiolite NC pattern showed a perfect match with the pattern obtained from PDF-2 database of ICCD (International Center for Diffraction Data) and the clinoptilolite NC pattern corresponded to a Nickel Ammonium Aluminium Silicon Hydroxide Oxide Hydrate (see Figure S1 in the Supplementary Material available online at http://dx.doi.org/10.1155/2015/164980). The chemical composition of nanoclays was determined by atomic absorption spectroscopy resulting in clinoptilolite nanoclays: 21.7% Na_2_O, 0.0078% MgO, 57.3% SiO_2_, 9.2% Al_2_O_3_, 1.4% CaO, 1% TiO_2_, 0.01% MnO, and for sepiolite nanoclays: 2.16% Na_2_O, 27.79% MgO, 37.15% SiO_2_, 12.01% Al_2_O_3_, 3.72% K_2_O, 3.92% CaO, 3.60% TiO_2_, and 7.22% MnO. In addition, the microanalysis of metallic elements and carbon was evaluated in the SEM micrographs (Figure S2). Results showed that 80% of clinoptilolite nanoparticles had a size down to 30 nm, with an average size of 17.5 nm, and 70% of sepiolite nanoparticles had a diameter size down to 20 nm. As this nanoclay is a fiber, fibers over 500 nm were also found (Figure 1).

### 3.2. Effect of Clinoptilolite and Sepiolite Nanoclays on* E. histolytica* Cultures

**Figure 2:**
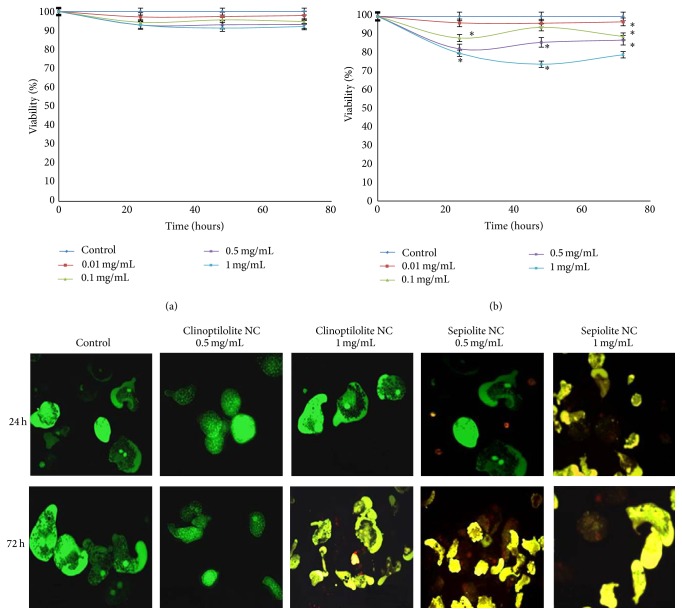
Viability of* E. histolytica* after the treatment with nanoclays. Amoebic cultures were treated with clinoptilolite (a) or sepiolite (b) NC for the period of time indicated and the viability measured by the Trypan blue method. Bottom pictures show CFDA/PI stained trophozoites from the treated cultures at the concentrations and time indicated. Controls are amoebas from a culture without treatment. The shifting of green to yellowish fluorescent indicates decrease of viability. Red nuclei come from dead cells.

The viability and growth of* E. histolytica* trophozoites were affected to different extents by the nanoclays. When treated with clinoptilolite NC, viability and growth were not significantly affected by incubation with any concentration at any time evaluated (Figure 2(a)). The integrity of trophozoites treated with clinoptilolite NC was confirmed on CFDA plus PI stained cells under fluorescence microscopy. In contrast, treatment with sepiolite NC, decreases the viability of trophozoites around 13 to 21% at 72 h with concentrations higher than 100 *μ*g/mL in a dose-dependent manner (*P* < 0.05 with respect to the untreated cultures). A slight recovery of amoebic viability was observed at 48 h for cultures treated with 100 and 500 *μ*g/mL; this recovery was apparent for cultures treated with 1000 *μ*g/mL until 72 h posttreatment (Figure 2(b)). The staining with CFDA plus PI showed that viability of amoebas seems to diminish over the time in a dose dependent manner, shifting from green to yellowish fluorescence, including red nuclei of death cells (Figure 2(c)).

**Figure 3:**
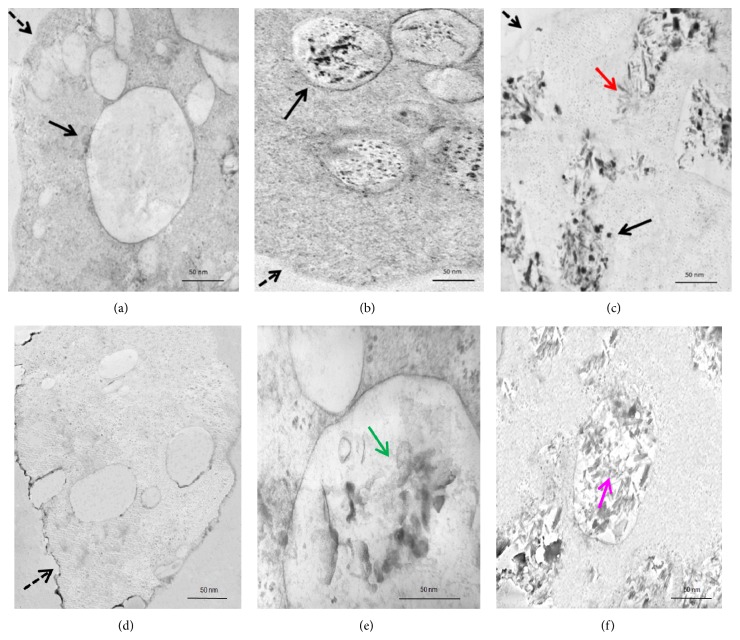
Uptake of nanoclays by amoebas and macrophages. TEM of 5 *μ*m thickness cross section of amoebas (a–c) and human macrophages (d–f) nontreated (a) and (d) or treated with clinoptilolite NC (b) and (e) or sepiolite NC (c) and (f). Dashed arrows are showing the cytosolic membrane of amoebas and macrophages. Black arrows are showing the vacuolar membrane, many of them containing high accumulation of clinoptilolite and sepiolite NC. Red arrow shows a possible vacuolar membrane rupture for sepiolite NC accumulation. The green and pink arrows show the accumulation of clinoptilolite and sepiolite NC in macrophages, respectively.

In order to demonstrate the uptake and cellular location of NC on treated cells, transmission electron microscopy (TEM) of* E. histolytica *trophozoites and human peripheral blood macrophages treated for 24 h with 100 *μ*g/mL of each NC was performed. Clinoptilolite NC were found dispersed in the cytosol and as aggregates inside the amoebic vacuoles (Figure 3(b)); in contrast, sepiolite NC were mainly found as aggregates inside large vacuoles and, in some cases, seem to cause the rupture of the vacuolar membrane (Figure 3(c), red arrow). In HMDM, clinoptilolite NC were observed inside phagocytic vacuoles larger than those observed in the amoebic cultures (Figure 2(e), green arrow); in the case of sepiolite NC, the distribution and size of the phagocytic vacuoles were similar to those of amoebas (Figure 3(f), pink arrow).

### 3.3. Effect of Clinoptilolite and Sepiolite NC on Macrophage Cultures

**Figure 4:**
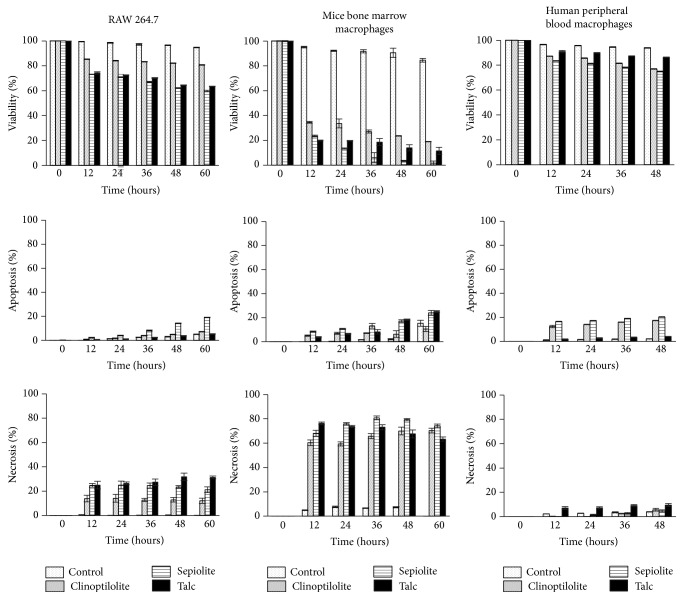
Viability, apoptosis, and necrosis of macrophages culture after treatment with nanoclays. Macrophages of RAW 264.7 cell line, from mice bone marrow monocytes and from human peripheral blood monocytes stained with Annexin V and propidium iodide to evaluate viability, apoptosis, and necrosis. Treatments with nanoclays and talc at 0, 12, 24, 36, 48 and 60 h are shown.

A dose and time dependent effect was observed on the viability of macrophages when treated with NC and talc, used here for comparing with the nanoparticle due to his long and proven history of safe use [31] as well as* in vitro* modest effect on peritoneal mouse macrophages [32]. Viability of RAW 264.7 macrophages decreased 15% at 24 h, reaching 20% at 60 h when treated with clinoptilolite NC, whereas sepiolite NC and talc affected the viability by 25% at 24 h to around 40% at 60 h (Figure 4; upper panels). HMDM showed a similar pattern but were less affected; in this case, clinoptilolite and sepiolite NC decreased the viability in around 25% and talc 14% at 60 h. However, a dramatic effect on the viability was rapidly observed in MMDM cultures, decreasing it to 65%, 73%, and 82% when treated with clinoptilolite NC, sepiolite NC, and talc at 24 h, respectively. The viability drops to 80%, 98%, and 88% at 60 h, respectively (Figure 4, upper panels). Cell death determined in the treated macrophage cultures using the Annexin V/propidium iodide kit showed that most RAW 264.7 and MMDM died by necrosis at all assay time, with no clear distinction between the different treatments (Figure 4, lower panels). In contrast, HMDM cultures, which were the least affected in viability, showed that about two thirds of the cells die by apoptosis at all times tested, suggesting that the cell processes that activate in macrophages the clinoptilolite NC are different than those activated by sepiolite NC and talc, leading to different outcomes (Figure 4, middle panels).

### 3.4. Cytokine Secretion Pattern from HMDM and MMDM Treated with NC

**Figure 5:**
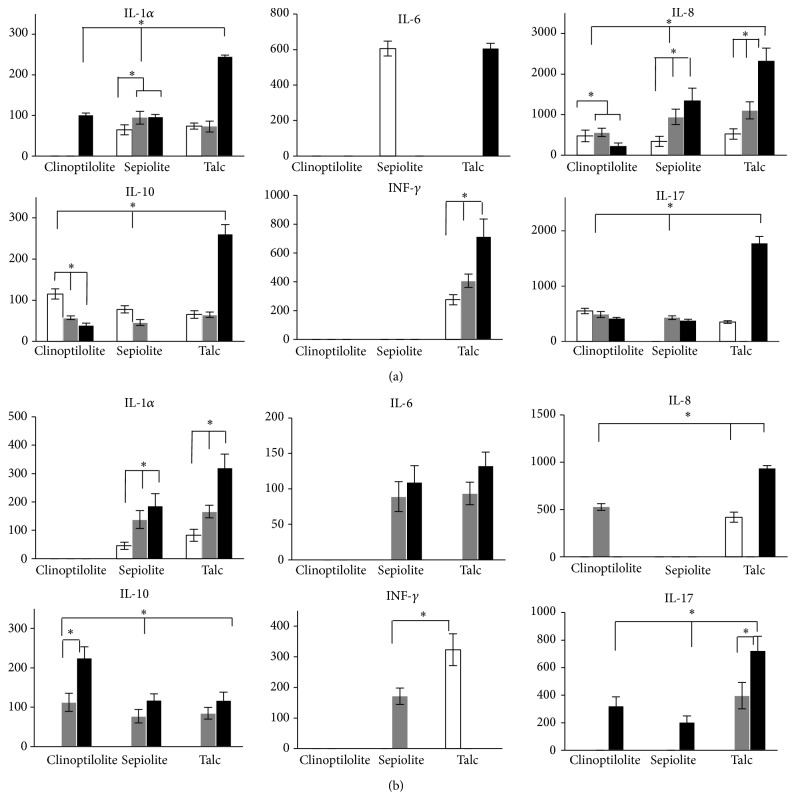
Cytokine secretion pattern from NC treated macrophages cultures. A panel of Th1 and Th2 cytokines was determined in the culture media of mice bone marrow macrophages (a) and human peripheral blood macrophages (b) at 24 h (open bars), 36 h (gray bars), and 48 h (black bars) posttreatment with NC and talc. All cytokine levels are shown in pg/mL ^*∗*^ *P* < 0.05.

The cytokines secreted by HMDM and MMDM (not RAW 264.7) in the presence of both NC and talc were determined in the culture media at 24, 36, and 48 h posttreatment by flow cytometry. Cytokines were undetectable at 12 h (data not shown) and were not determined at 60 h. In general terms, proinflammatory cytokines increased over the time in the two types of macrophages, but at different levels depending on treatments (Figure 5). A proinflammatory profile was clearer with sepiolite NC and the talc treatments, compared with clinoptilolite NC treatment. Thus, in HMDM treated with sepiolite NC or talc, some amounts of IL-1*α* and IL-6 were detected that slightly increased over the time. In contrast, release of IL-6 and INF*γ* was not detected in neither MMDM nor HMDM treated with clinoptilolite at any time evaluated (Figures 5(a) and 5(b), resp.). The cytokine/chemokine IL-8 was released without a clear pattern by any treatment, but its production was considerably higher in MMDM treated with sepiolite NC or talc, compared with clinoptilolite NC (*P* < 0.05) (Figure 5(a)). Few levels of this cytokine were detected in medium from HMDM treated. The other cytokine highly expressed was IL-17, which appeared under any treatment at different times, with its production being higher in MMDM and HMDM treated with talc. INF*γ* was increasingly induced only by talc in MMDM and by sepiolite NC and talc in HMDM, but not by clinoptilolite NC. GM-CSF and Th2 cytokines IL-4 and IL-5 were not detected under any condition tested (not shown). Low levels of the regulatory cytokine IL-10 were detected in media from treated MMDM (Figure 5(a)) in contrast with high levels detected in HMDM treated with clinoptilolite NC with respect to sepiolite NC and talc (*P* < 0.05) (Figure 5(b)).

## 4. Discussion

Clinoptilolite and sepiolite have been proposed for pharmaceutical applications including tablet manufacture [33], slow release systems [34], in combination with drugs for cancer therapy [35], being as adjuvants [36], being as adsorbent trapping lead in children with ADHD by intravenous administration [5], and, in general terms, for diverse therapy in humans [37]. In this sense, the development of nanoparticles as drug delivery systems increased the interest in nanoclays [3–8] due to their physical and chemical properties in comparison to the natural clays. However, even if the toxicity of microsized clays has been evaluated [38, 39], the International Agency for Research on Cancer reports that there are few reports regarding* in vitro* and* in vivo* clinoptilolite and sepiolite NC biocompatibility [24]. Thereby, in this work we evaluated the cytotoxic effect of clinoptilolite and sepiolite NC in two of the most highly phagocytic cells reported [27–29], as a first approach to determining nanoclays biocompatibility. One of the precautions that need to be taken into account for* in vitro* tests with clinoptilolite is that this nanoclay could modify the ion composition of the culture media and thereof mask the toxicity of this nanoclay [40]. In order to prevent this, nanoclay suspensions stocks were prepared in supplemented TYI-S-33 and DMEM culture medium, exposing the nanoclays to serum and glucose prior to its addition to the experimental cultures decreasing the probability of affecting the cellular cultures by the ion-exchange features of NC. In addition, the nanoclays suspensions were sonicated in order to reduce any possible unspecific effect of the NC aggregation. With this treatments we assumed that ion composition of the medium and dispersion ratio of NC was nearly constant during the experiments, so neither of the two is the principal cause of cellular death in our cultures.

Our results showed that even when both NC were highly phaghocytosed by amoebas, clinoptilolite NC was not toxic to* E. histolytica* trophozoites, in contrast to sepiolite NC that showed significant cytotoxic effect, suggesting that clinoptilolite is less toxic than sepiolite NC, at least against the parasite. As the sepiolite NC-treated trophozoites did not show evidence of lysis or significant morphological changes, we think that amoebas probably die by an apoptotic process, a type of cellular death known that occurs in amoeba [41] (Figure 2). However, apoptosis of amoeba was not analyzed in this work and should be conducted in further studies.

On the other hand, viability of macrophages cultures was affected at different extents, but in a dose and time dependent manner by the NC. Even when the viability of the macrophages was affected in a larger extent than the* E. histolytica* trophozoites, in agreement with the results obtained with amoebas, clinoptilolite NC were less toxic than sepiolite NC in all the macrophage cultures tested, supporting clinoptilolite NC as more biocompatible. However, sepiolite NC treatment showed a comparable effect with asbestos-free talc powder used as nanoparticle compound with a proven long history of safe use [31], suggesting that even when sepiolite NC is more cytotoxic than clinoptilolite NC, both are relatively harmless. Sohaebuddin et al. [22] have reported that a different type of cells has a different cytotoxic response against nanoparticles. Remarkably, we observed in this study that the cytotoxic effect also depends on the cell origin, with the MMDM cultures being the most affected (more than 80% of viability reduction at 60 h) followed by RAW 264.7 and finally HMDM (Figure 4). The RAW 264.7 cell line has widely been used for analyzing cytotoxicity of silica, polymers, metal oxides, silver, and gold nanoparticles [42–45], including two reports on hydroxyapatite and boehmite NC [46, 47], showing higher susceptibility than the results obtained here with the NC. Thus, our results suggest that not only the type but also the origin of the cell should be taken into account when testing cytotoxicity of NC, which could be extensive to any nanoparticle. However, we cannot rule out that other factors can influence the results and therefore, such proposal needs to be confirmed using a wide variety of cells from different sources.

In this work, the results obtained in the RAW 264.7 cell line macrophages were similar to the effect reported for montmorillonite NC on the human hepatic cell line HepG2, where cell viability was reduced in more than 20% with the same dose of 1 mg/mL [48]. Even when different cell lines were used, clinoptilolite NC and montmorillonite NC showed a similar effect on viability probably due to their structure [49]. However, we cannot rule out the possibility of differences associated with the particular characteristics of each cell line. Remarkably, HMDM cultures were the less affected by the treatments with NC or talc, never showing more than 25% of viability reduction at the time and doses tested (Figure 4). This is particularly interesting if we consider that potential biomedical applications in humans of NC will lead in any point to the encounter of the nanoparticles with the highly phagocytic scavenger macrophage, and its use is highly recommended by the international standard ISO 10993-5 for the biological evaluation of medical devices. In this sense, HMDM has been used to evaluate the toxicity of many particles present in dust, polluted air, polymers, and others [50–52]. However, in our knowledge, HMDM cultures have only been used to assess the cytotoxicity of MWCNTs and nanoparticles of titanium and zinc metal oxide, but not nanoclays [53, 54].

Interestingly, uptakes of both NC by amoebas and HMDM appear to be similar, due to their location inside large phagocytic vesicles or lysosomes, suggesting a phagocytic or macropinocytic process (Figure 3). In this sense, the greater aggregation of sepiolite NC inside larger vesicles could affect the vacuolar membrane compared to the less aggregation of clinoptilolite NC, which could be also related with the higher cytotoxic effect of sepiolite NC on the cultures. As the toxicity of nanoparticles depends on the size and composition of the material [22] and the aggregation state is associated with the NC structure, the nanofiber structure of sepiolite NC could aggregate and puncture the vesicle membrane more easily than the deformed octahedron clinoptilolite NC, explaining the higher toxic effect of sepiolite NC.

Regarding the type of cell death induced by the NC, noteworthy, the predominant cell death was different depending on the macrophage culture studied. Thus, RAW 264.7 and MMDM cultures mainly died by necrosis (two- and threefold over the number of apoptotic cells, resp.), whereas HMDM preferably died by an apoptotic pathway (twofold over the number of necrotic cells). HMDM death could be comparable with amoebic cultures, where the morphology of the death trophozoites suggests an apoptotic process. The mechanisms triggering the macrophage death by the NC and talc as well as the underlying signaling events leading to apoptosis or necrosis are unknown, but they could involve surface scavenger receptors and activation of mitochondrial caspase 9 as described for the toxic effect of zinc oxide nanoparticles [55].

In terms of cytotoxicity, apoptosis death could be more preferable than necrosis, due to the potential of necrotic cellular debris to promote a proinflammatory response that is associated with tissue damage [56]. The proinflammatory response involves the release by the macrophages and other innate cells of cytokines and chemokines that promote recruitment of new cells to the site of infection or damage. Therefore, the cytokine secretion pattern in the supernatant of primary culture macrophages (MMDM and HMDM) treated with the NC and talc was analyzed by flow cytometry. As expected, a correlation between the cytokine patterns released by the NC exposed macrophages and the observed cellular death pathways was found. Thus, the MMDM cultures, which were the most affected by the treatments and mainly dying by necrosis, secreted higher levels of proinflammatory cytokines Il-1*α*, IL-8, and IL6, in comparison with HMDM cultures. In agreement with our results, it has been reported that human macrophages/monocytes stimulated with single-walled carbon nanotubes (CNT) or silica induced the release of IL-1*α*, IL-6, and IL-8 associated with a proinflammatory outcome [53]; also, the application of natural clinoptilolite in mice food for 28 days produced an increased serum LSA concentration which could be related with the release of TNF-*α* and IL-1 by macrophages [57]. As mentioned before, MMDM cultured with NC released higher amount of proinflammatory cytokines than HMDM, mainly IL-8 and IL-6 (Figure 5). In addition, the release of IL-8 in MMDM was higher with sepiolite NC than with clinoptilolite NC. The induction of some of these proinflammatory cytokines by the NC could be related to their agglomeration state [58] and to the particle size, the bigger the particle the higher release [59]. Therefore, the higher IL-8 release from MMDM in the presence of sepiolite NC could be related to the bigger size of sepiolite NC in comparison with clinoptilolite NC.

This was also observed for INF*γ*, another important proinflammatory cytokine, which was only detected in two time points of HMDM treatment with sepiolite NC and talc (36 h and 24 h postexposure, resp.), but not with clinoptilolite NC (Figure 5). On the other hand, TNF*α* was undetectable in the macrophage cultures with any NC or talc (not shown), suggesting low toxicity as the most biocompatible materials have been shown to induce low TNF-*α* levels that tend to drop to zero over the time [60]. Moreover, the expression of cytokine IL-17 at late time (48 h) suggests that NC and talc have the potential to induce an allergic response, as it has been described in alveolar macrophages of animals orally treated with PLGA NPs coated with chitosan and PEG [61]. In addition to the proinflammatory pattern of cytokines, HMDM cultures release higher amount of IL-10 than MMDM cultures in the presence of NC, a regulatory cytokine probably produced to counterbalance the proinflammatory profile. Interestingly, the expression of IL-10 has been associated with a greater biocompatibility contributing to the inhibition or resolution of the inflammation associated with nanocomposites [59], which could be related with the lower toxicity of NC on HMDM.

The results of this work showed that clinoptilolite and sepiolite NC are well tolerated when tested in highly phagocytic cell cultures, showing results comparable with asbestos-free talc powder suggesting that both could be highly biocompatible. However, when compared, clinoptilolite NC appears to be less toxic than sepiolite NC, which is very important taking into account the potential biomedical application of clinoptilolite in humans. These cytotoxic assays could contribute to the necessary knowledge for future application of nanoclays; however, additional studies regarding the cellular physiology alterations of cells from different lineages as well as* in vivo* studies at short and long term exposure to confirm the safety of clinoptilolite and sepiolite nanoclays are necessary before thinking in their use for biomedical applications.

## Supplementary Material

In order to characterize the samples of clinoptilolite and sepiolite NC used in this work, besides the atomic absorption analysis shown in the results section, the pattern of X-ray diffraction and microanalysis performed by SEM were obtained for both samples. Figure S1 shows the peaks corresponding to diffraction planes obtained for clinoptilolite NC (top graph) and sepiolite NC (bottom graph), which when compared with the PDF-2 database from the International Centre for Diffraction Data, match with the data reported for clinoptilolite-Ni (Nickel Aluminium ammonium hydroxide hydrated silicon oxide) and sepiolite, respectively. Figure S2 shows the results of microanalysis performed by SEM of clinoptilolite NC (top graph) and sepiolite NC (bottom graph), which confirm their identity.

## References

[1] Charkhi A., Kazemian H., Kazemeini M. (2010). Optimized experimental design for natural clinoptilolite zeolite ball milling to produce nano powders. *Powder Technology*.

[2] Can M. F., Çinar M., Benli B., Özdemir O., Çelik M. S. (2010). Determining the fiber size of nano structured sepiolite using Atomic Force Microscopy (AFM). *Applied Clay Science*.

[3] Hrenovic J., Milenkovic J., Daneu N., Kepcija R. M., Rajic N. (2012). Antimicrobial activity of metal oxide nanoparticles supported onto natural clinoptilolite. *Chemosphere*.

[4] Zayed M. A., El-Begawy S. E. M., Hassan H. E. S. (2012). Enhancement of stabilizing properties of double-base propellants using nano-scale inorganic compounds. *Journal of Hazardous Materials*.

[5] Delavarian M., Hassanvand A., Gharibzadeh S. (2013). Increasing performance in children with ADHD by trapping lead with a nano-zeolite. *Journal of Neuropsychiatry and Clinical Neurosciences*.

[6] Nezamzadeh-Ejhieh A., Kabiri-Samani M. (2013). Effective removal of Ni(II) from aqueous solutions by modification of nano particles of clinoptilolite with dimethylglyoxime. *Journal of Hazardous Materials*.

[7] Barrera-Díaz C., Almaraz-Calderón C., Olguín-Gutiérrez M. T., Romero-Romo M., Palomar-Pardavé M. (2005). CD(II) and PB(II) separation from aqueous solution using clinoptilolite and *Opuntia* ectodermis. *Environmental Technology*.

[8] Xie S., Zhang S., Wang F., Yang M., Séguéla R., Lefebvre J.-M. (2007). Preparation, structure and thermomechanical properties of nylon-6 nanocomposites with lamella-type and fiber-type sepiolite. *Composites Science and Technology*.

[9] Ha J. U., Xanthos M. (2011). Drug release characteristics from nanoclay hybrids and their dispersions in organic polymers. *International Journal of Pharmaceutics*.

[10] Ray S. S. (2012). Polylactide-based bionanocomposites: a promising class of hybrid materials. *Accounts of Chemical Research*.

[11] Yang M., Wang P., Huang C. Y., Ku M. S., Liu H., Gogos C. (2010). Solid dispersion of acetaminophen and poly(ethylene oxide) prepared by hot-melt mixing. *International Journal of Pharmaceutics*.

[12] Campbell K., Craig D. Q. M., McNally T. (2008). Poly(ethylene glycol) layered silicate nanocomposites for retarded drug release prepared by hot-melt extrusion. *International Journal of Pharmaceutics*.

[13] Choi S.-J., Choy J.-H. (2011). Layered double hydroxide nanoparticles as target-specific delivery carriers: uptake mechanism and toxicity. *Nanomedicine*.

[14] Campbell K. T., Craig D. Q. M., McNally T. (2010). Ibuprofen-loaded poly(e-caprolactone) layered silicate nanocomposites prepared by hot melt extrusion. *Journal of Materials Science: Materials in Medicine*.

[15] Solhi L., Atai M., Nodehi A., Imani M., Ghaemi A., Khosravi K. (2012). Poly(acrylic acid) grafted montmorillonite as novel fillers for dental adhesives: synthesis, characterization and properties of the adhesive. *Dental Materials*.

[16] Ambre A. H., Katti D. R., Katti K. S. (2013). Nanoclays mediate stem cell differentiation and mineralized ECM formation on biopolymer scaffolds. *Journal of Biomedical Materials Research A*.

[17] Feng R., Zhang Y., Li H. (2013). Ultrasensitive electrochemical immunosensor for zeranol detection based on signal amplification strategy of nanoporous gold films and nano-montmorillonite as labels. *Analytica Chimica Acta*.

[18] Kipen H. M., Laskin D. L. (2005). Smaller is not always better: nanotechnology yields nanotoxicology. *American Journal of Physiology: Lung Cellular and Molecular Physiology*.

[19] Powell M. C., Kanarek M. S. (2006). Nanomaterial health effects—part 2: uncertainties and recommendations for the future. *Wisconsin Medical Journal*.

[20] Thomas K., Sayre P. (2005). Research strategies for safety evaluation of nanomaterials. Part I. Evaluating the human health implications of exposure to nanoscale materials. *Toxicological Sciences*.

[21] Oberdörster G., Oberdörster E., Oberdörster J. (2005). Nanotoxicology: an emerging discipline evolving from studies of ultrafine particles. *Environmental Health Perspectives*.

[22] Sohaebuddin S. K., Thevenot P. T., Baker D., Eaton J. W., Tang L. (2010). Nanomaterial cytotoxicity is composition, size, and cell type dependent. *Particle and Fibre Toxicology*.

[23] Magdolenova Z., Collins A., Kumar A., Dhawan A., Stone V., Dusinska M. (2014). Mechanisms of genotoxicity. A review of *in vitro* and *in vivo* studies with engineered nanoparticles. *Nanotoxicology*.

[24] IARC (2013). Arsenic, metals, fibres, and dusts. *IARC Working Group on the Evaluation of Carcinogenic Risk to Humans*.

[25] Banquy X., Suarez F., Argaw A. (2009). Effect of mechanical properties of hydrogel nanoparticles on macrophage cell uptake. *Soft Matter*.

[26] Moghimi S. M., Hunter A. C., Murray J. C. (2005). Nanomedicine: current status and future prospects. *The Faseb Journal*.

[27] Service R. F. (2004). Nanotechnology grows up. *Science*.

[28] Vergaro V., Abdullayev E., Lvov Y. M. (2010). Cytocompatibility and uptake of halloysite clay nanotubes. *Biomacromolecules*.

[29] Sateriale A., Vaithilingam A., Donnelly L., Miller P., Huston C. D. (2012). Feed-forward regulation of phagocytosis by *Entamoeba histolytica*. *Infection and Immunity*.

[30] Carrero-Sánchez J. C., Elías A. L., Mancilla R. (2006). Biocompatibility and toxicological studies of carbon nanotubes doped with nitrogen. *Nano Letters*.

[31] Zazenski R., Ashton W. H., Briggs D. (1995). Talc: occurrence, characterization, and consumer applications. *Regulatory Toxicology and Pharmacology*.

[32] Khan M. I., Sahasrabuddhe A. A., Patil G., Akhtar M. J., Ashquin M., Ahmad I. (2011). Nano-talc stabilizes TNF-*α* m-RNA in human macrophages. *Journal of Biomedical Nanotechnology*.

[33] Viseras C., Lopez-Galindo A. (1999). Pharmaceutical applications of some Spanish clays (sepiolite, palygorskite, bentonite). *Applied Clay Science*.

[34] Andronescu E., Grigore F., Tardei C., Stefan E. (2006). Natural zeolites with medical applications—preliminary preparation and characterization. *Revista Medico: Chirurgicala a Societatii de Medici si Naturalisti din Iasi's*.

[35] Zarkovic N., Zarkovic K., Kralj M. (2003). Anticancer and antioxidative effects of micronized zeolite clinoptilolite. *Anticancer Research*.

[36] Pavelić K., Hadžija M., Bedrica L. (2001). Natural zeolite clinoptilolite: new adjuvant in anticancer therapy. *Journal of Molecular Medicine*.

[37] Jurkić L. M., Cepanec I., Pavelić S. K., Pavelić K. (2013). Biological and therapeutic effects of ortho-silicic acid and some ortho-silicic acid-releasing compounds: new perspectives for therapy. *Nutrition and Metabolism*.

[38] Adamis Z., Tátrai E., Honma K., É S., Ungváry G. (2000). *In vitro* and *in vivo* tests for determination of the pathogenicity of quartz, diatomaceous earth, mordenite and clinoptilolite. *The Annals of Occupational Hygiene*.

[39] Elmore A. R. (2003). Final report on the safety assessment of aluminum silicate, calcium silicate, magnesium aluminum silicate, magnesium silicate, magnesium trisilicate, sodium magnesium silicate, zirconium silicate, attapulgite, bentonite, Fuller's earth, hectorite, kaolin, lithium magnesium silicate, lithium magnesium sodium silicate, montmorillonite, pyrophyllite, and zeolite. *International Journal of Toxicology*.

[40] Katic M., Bosnjak B., Gall-Troselj K., Dikic I., Pavelic K. (2006). A clinoptilolite effect on cell media and the consequent effects on tumor cells *in vitro*. *Frontiers in Bioscience*.

[41] Ghosh A. S., Dutta S., Raha S. (2010). Hydrogen peroxide-induced apoptosis-like cell death in *Entamoeba histolytica*. *Parasitology International*.

[42] Hamada T., Morita M., Miyakawa M. (2012). Size-dependent partitioning of nano/microparticles mediated by membrane lateral heterogeneity. *Journal of the American Chemical Society*.

[43] Chen B., Liu Y., Song W. M., Hayashi Y., Ding X. C., Li W. H. (2011). *In vitro* evaluation of cytotoxicity and oxidative stress induced by multiwalled carbon nanotubes in murine RAW 264.7 macrophages and human A549 Lung cells. *Biomedical and Environmental Sciences*.

[44] Tripathy N., Hong T.-K., Ha K.-T., Jeong H.-S., Hahn Y.-B. (2014). Effect of ZnO nanoparticles aggregation on the toxicity in RAW 264.7 murine macrophage. *Journal of Hazardous Materials*.

[45] Elsabahy M., Zhang S., Zhang F. (2013). Surface charges and shell crosslinks each play significant roles in mediating degradation, biofouling, cytotoxicity and immunotoxicity for polyphosphoester-based nanoparticles. *Scientific Reports*.

[46] Orlowski P., Krzyzowska M., Zdanowski R. (2013). Assessment of *in vitro* cellular responses of monocytes and keratinocytes to tannic acid modified silver nanoparticles. *Toxicology in Vitro*.

[47] Zhang T., Tang M., Kong L. (2012). Comparison of cytotoxic and inflammatory responses of pristine and functionalized multi-walled carbon nanotubes in RAW 264.7 mouse macrophages. *Journal of Hazardous Materials*.

[48] Lordan S., Kennedy J. E., Higginbotham C. L. (2011). Cytotoxic effects induced by unmodified and organically modified nanoclays in the human hepatic HepG2 cell line. *Journal of Applied Toxicology*.

[49] Martin R. T., Bailey S. W., Eberl D. D. (1991). Report of the clay minerals society nomenclature committee: revised classification of clay materials. *Clays and Clay Minerals*.

[50] Lombaert N., Lison D., van Hummelen P., Kirsch-Volders M. (2008). *In vitro* expression of hard metal dust (WC-Co) responsive genes in human peripheral blood mononucleated cells. *Toxicology and Applied Pharmacology*.

[51] Pettit A. P., Brooks A., Laumbach R. (2012). Alteration of peripheral blood monocyte gene expression in humans following diesel exhaust inhalation. *Inhalation Toxicology*.

[52] Bosetti M., Zanardi L., Bracco P., Costa L., Cannas M. (2003). *In vitro* evaluation of the inflammatory activity of ultra-high molecular weight polyethylene. *Biomaterials*.

[53] Laverny G., Casset A., Purohit A. (2013). Immunomodulatory properties of multi-walled carbon nanotubes in peripheral blood mononuclear cells from healthy subjects and allergic patients. *Toxicology Letters*.

[54] Tuomela S., Autio R., Buerki-Thurnherr T. (2013). Gene expression profiling of immune-competent human cells exposed to engineered zinc oxide or titanium dioxide nanoparticles. *PLoS ONE*.

[55] Wilhelmi V., Fischer U., Weighardt H. (2013). Zinc oxide nanoparticles induce necrosis and apoptosis in macrophages in a p47phox- and Nrf2-independent manner. *PLoS ONE*.

[56] Guo X., Jagannath C., Espitia M. G., Zhou X. (2012). Uptake of silica and carbon nanotubes by human macrophages/monocytes induces activation of fibroblasts *in vitro*—potential implication for pathogenesis of inflammation and fibrotic diseases. *International Journal of Immunopathology and Pharmacology*.

[57] Pavelic K., Katic M., Sverko V. (2002). Immunostimulatory effect of natural clinoptilolite as a possible mechanism of its antimetastatic ability. *Journal of Cancer Research and Clinical Oncology*.

[58] Schanen B. C., Das S., Reilly C. M. (2013). Immunomodulation and T helper TH1/TH2 response polarization by CeO_2_ and TiO_2_ nanoparticles. *PLoS ONE*.

[59] Oh W.-K., Kim S., Choi M. (2010). Cellular uptake, cytotoxicity, and innate immune response of silica—titania hollow nanoparticles based on size and surface functionality. *ACS Nano*.

[60] Heuking S., Rothen-Rutishauser B., Raemy D. O., Gehr P., Borchard G. (2013). Fate of TLR-1/TLR-2 agonist functionalised pDNA nanoparticles upon deposition at the human bronchial epithelium *in vitro*. *Journal of Nanobiotechnology*.

[61] Semete B., Booysen L. I. J., Kalombo L. (2010). *In vivo* uptake and acute immune response to orally administered chitosan and PEG coated PLGA nanoparticles. *Toxicology and Applied Pharmacology*.

