# Supplementary material for: Effect of Clinoptilolite and Sepiolite Nanoclays on Human and Parasitic Highly Phagocytic Cells
Source: Biomed Res Int. 2015 May 27;2015:164980. doi: 10.1155/2015/164980 (PMC4452243; doi:10.1155/2015/164980)
Supplement: Supplementary file 1 — In order to characterize the samples of clinoptilolite and sepiolite NC used in this work, besides the atomic absorption analysis shown in the results section, the pattern of X-ray diffraction and microanalysis performed by SEM were obtained for both samples. Figure S1 shows the peaks corresponding to diffraction planes obtained for clinoptilolite NC (top graph) and sepiolite NC (bottom graph), which when compared with the PDF-2 database from the International Centre for Diffraction Data, match with the data reported for clinoptilolite-Ni (Nickel Aluminium ammonium hydroxide hydrated silicon oxide) and sepiolite, respectively. Figure S2 shows the results of microanalysis performed by SEM of clinoptilolite NC (top graph) and sepiolite NC (bottom graph), which confirm their identity. [file 164980.f1.pdf]

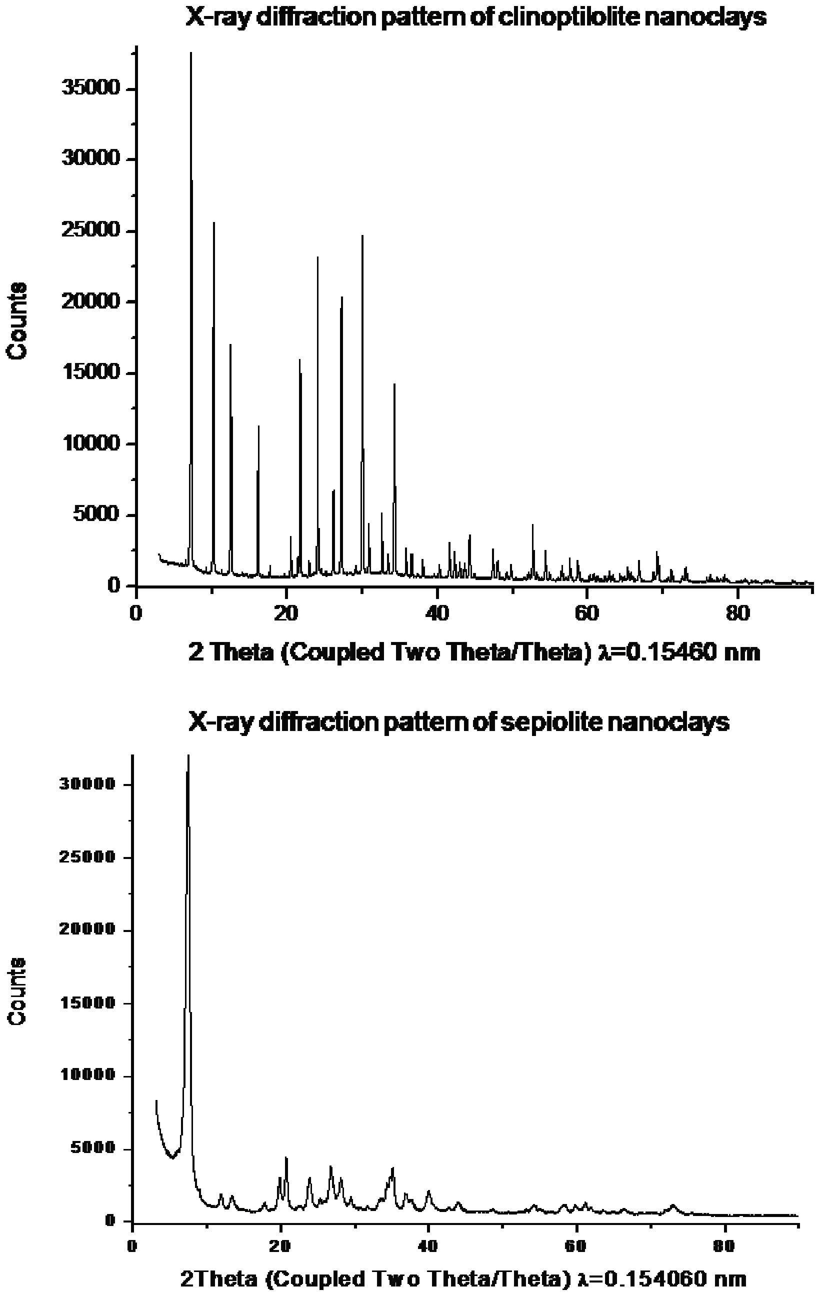

Figure S1. X-ray diffraction pattern of clinoptilolite and sepiolite nanoclays. The pics correspond to the diffraction planes. Sepiolite nanoclays match perfectly with the pattern of the PDF-2 database from the International Centre for Diffraction data and Clinoptilolite NC correspond to a Nickel Ammonium Aluminium Silicon Hydroxide Oxide Hydrate.

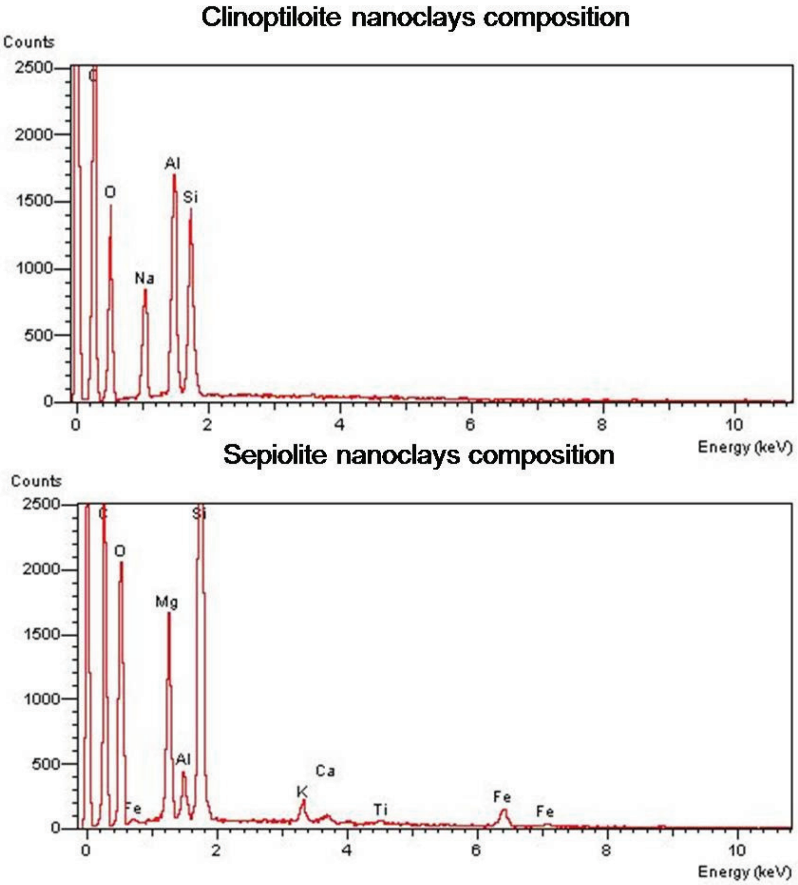

Figure S2. Microanalysis of clinoptilolite and sepiolite nanoclays determined by SEM. Metal content of nanoclays was the same that data found by atomic absorption.
